# Modern Sociology

**Published:** 1903-09-05

**Authors:** 


### MODERN SOCIOLOGY.

### PEAT WATER AND LEAD POISONING.

THE existence of cases of lead poisoning, where there was nothing apparent in the employment nor environment of the sufferers to cause it, led sanitarians to consider the possibility of the source of infection being the actual water supply of the neighbourhood. Water containing a certain proportion of acid has the power of more or less dissolving the lead pipes through which it is conveyed to a distance, and thus water which, so far as the actual acid is concerned, is harmless enough, becomes dangerous to health through the action of this acid on the leaden pipes. This acidity is common in the water of moorland gathering grounds, because of the peaty soil through which it passes, and with a view to investigating the matter, the President of the Local Government Board asked Dr. Houston to study these waters, determine the amount of plumbo-solvency possessed by different specimens, and suggest means whereby this power could be neutralised.

Dr. Houston analysed the water supply of twenty-three gathering grounds in Lancashire and Yorkshire, and with the assistance of local experts made a weekly analysis of the water of the Bentham waterworks, taking note not only of the mixed product contained in the reservoir, but of the various springs and streams from which the supply there was derived. Bentham was chosen because, although it was a small gathering ground in itself, it was in every way typical of the larger ones in Lancashire and Yorkshire. The lengthened and periodic analysis was undertaken in order to discover three things:—What were the seasonal modifications of each of the contributory waters; in what way and to what degree they differed at one time and another from each other as regards their constituents; and how far their ability to dissolve lead was associated with the season of the year. The importance of the lengthened period of observation is made clear when it is found that water from the same source of supply which in December showed a degree of plumbo-solvency indicated by the figures 0·160, and in the middle of February of 0·136 proved to be under the same tests absolutely free from such power in the middle of March. It might be thought that colour and clearness would be an indication of the good or bad quality of the water. But this is not so. One of the worst specimens as regards plumbo-solvency was clear and colourless, while of the two which showed absolutely no such power under analysis, one was straw-coloured and one bluish. The different waters of the same gathering ground, which meet and mingle in the reservoir may be and often are of very different character, some of them acid and therefore more or less able to dissolve lead, while the others are alkaline and have a counteracting power. Thus the water which comes from the reservoir may be practically unable to affect the pipes through which it flows, although some of its constituents are perceptibly acid. If the supply were then always equal and constant, there might be little danger to health, even though some of the contributory waters to a given supply were acid. But the varying rainfall causes a variety in the mixture in the reservoir. In dry weather, the neutral or alkaline water derived from springs will be proportionately larger, and the general product safe, while a flood which sends down more surface water which has flowed through peaty ground into the reservoir will largely alter its characteristics. Unhappily, a small amount of peat can render acid a relatively large bulk of neutral water, and the peaty water is subject to far larger variations of quantity than the neutral spring water. On the other hand, the degree of acidity in different samples of peaty water varies a good deal, and it is therefore not possible to say definitely what amount of surface water coming from peaty ground will destroy the alkalinity of a given quantity of spring water. In moorland gathering grounds the amount of spring water is often relatively small, because these grounds lie so high. Much of the water that sinks deeply into the earth crops out at a lower level than that of most waterworks. This may account for the fact that, in Sheffield, that part of the population which was supplied with water from a high-level system was subject to lead poisoning, while those whose water supply came from a low-level system escaped.

The acidity of the water is affected to a considerable extent by the slope of the hills down which the streams flow. Usually there is found under peat a layer of marl. This marl has a certain counteracting effect on acid water. But water does not benefit much by merely lying on either of these, or flowing slowly over them on a gentle slope. Where, however, the stream runs through a steep gorge, and considerable denudation of the underlying rock is constantly going on, the disintegrated sand or rock will have a neutralising effect on the acidity of the water. But where the ground is flat and the water lies for some time becoming saturated with peat the degree of acidity will be high. A heavy rainfall which largely increases the flow of peaty streams might be expected at least to weaken the acidity at the same time, but if the ground below the peat be impervious clay, the rain rather forces out into the streams which feed the reservoir, water which has for some time been lying stagnant in the moss and becoming saturated with peat, and the fresh rain takes the place of this, to become tainted in turn.

With regard to the prevention of the dangers incident to the drinking of this water, the best is, of course, to avoid as far as possible the use of waters which have been rendered acid by passing through peat. It is not possible, however, for towns which must depend for their water-supply on the moorland streams in their neighbourhood to eliminate altogether water which is thus made acid. They must depend on the treatment of such water as they can get. There are contrivances called "leaping weirs" which ensure that the first washings from the peat, after a period of drought, will be automatically rejected. By providing these in many cases the peat water would not be more acid than could be corrected by the natural admixture of neutral or alkaline spring water. Failing this, much can be done by proper filtration at the waterworks. Dr. Houston thinks that the most satisfactory method is that of ordinary sand filtration with some neutralising material added, such as a limestone base under the sand and a thin coating of lime on top of it. After the water has been thus filtered a trace of sodium carbonate may be added to it. Thus treated, the water will lose its power of dissolving lead. Doubtless the authorities of towns will not neglect these means of making their water supply wholesome. To make sure of this, however, it is necessary that frequent observations should be made on the gathering grounds, at the reservoirs, and at the filters, so that any failure in their efficacy may be at once noted. The water as it flows through the consumers' taps should also be tested occasionally, so that watch may be kept at every point. Otherwise those occasional mysterious cases of lead poisoning which at present appear will not entirely vanish.

